# Early versus delayed laparoscopic common bile duct exploration for common bile duct stone-related nonsevere acute cholangitis

**DOI:** 10.1038/srep11748

**Published:** 2015-06-30

**Authors:** Bin Zhu, Dan Li, Yu Ren, Ying Li, Yan Wang, Kai Li, Buhe Amin, Ke Gong, Yiping Lu, Ming Song, Nengwei Zhang

**Affiliations:** 1 Laparoscopic Surgical Center, Department of General Surgery of Beijing Shijitan Hospital, Capital Medical University. Tieyilu 10, Yangfangdian, Haidian District, Beijing 100038, P.R. China; 2 Department of medicine, Division of Gastroenterology, Hepatology and Nutrition, University of Louisville School of Medicine, Louisville, Kentucky, US

## Abstract

It is undetermined when and how laparoscopic common bile duct exploration (LCBDE) should be used in patients with common bile duct (CBD) stone-related nonsevere acute cholangitis. We aimed to evaluate the effect of LCBDE on the clinical outcome of those patients within (early) or beyond (delayed) 72 hours of emergent admission. Surgery-related complications, length of hospital stay (LOS), and total cost, as well as demographic and clinical parameters were compared between the two groups. Finally, 3 and 5 patients in early and delayed LCBDE group, respectively, had retained stones, which were removed by choledochoscopy before T-tube was removed. Each group had 3 patients who developed biliary leak, which was conservatively cured by the drainage. Shorter LOS and less total cost were observed in early group compared to the late one (13.34 ± 4.48 vs. 18.32 ± 9.13, *p* < 0.05; 17712 ± 5446.63 vs. 21776 ± 7230.41 ¥RMB, *p* < 0.05). Improvement of cholangitis was achieved in all patients with LCBDE. None of the patients developed stricture of the CBD after LCBDE. To conclude, both early and delayed LCBDE are safe and effective for the treatment of CBD stone-related nonsevere acute cholangitis during emergent admissions. Early LCBDE may be superior to delayed procedure due to the shorter LOS and less cost.

Gallstones are present in approximately 15% of the US population. Whilst figures quoted vary according to the age, sex and ethnicity of the group examined, the overall prevalence in the United Kingdom is likely to be similar^1^. Common bile duct (CBD) stones may occur in 3%–14.7% of all patients for whom cholecystectomies are preformed. The primary etiology of acute cholangitis is the presence of stones^2^. The incidence proportion of the appearance of acute cholangitis in patients with gallstones is 0.3%–1.6%. Acute cholangitis is a morbid condition with acute inflammation and infection in the bile duct^3^. The generally used guideline for the diagnosis, severity assessment and treatment of acute cholangitis is “Tokyo Guidelines”^3^,^4^,^5^,^6^,^7^. The “Guidelines” recommended a more systematic approach, using a combination of clinical features, laboratory data, and imaging findings to diagnose acute cholangitis. In the current study, we followed “Tokyo Guidelines” diagnostic criteria for acute cholangitis. Severe acute cholangitis is defined as acute cholangitis that is associated with the onset of dysfunction in at least one of any of the organs/systems^3^,^4^. The proportion of cases diagnosed as severe acute cholangitis was 12.3% of acute cholangitis due to bile duct stones^8^. Patients with acute cholangitis are at risk of developing severe and potentially lethal infections such as sepsis unless appropriate medical care is provided promptly. It has been reported that the mortality rate of acute cholangitis was higher than 50% before 1980 and 10%–30% in 1981–1990s. Despite the advancement in diagnosis and management, the mortality remains about 10% after 2000^8^. As a therapeutic procedure for severe cases or to prevent increased severity, decompression of the biliary tract (i.e., biliary tract drainage) is necessary. The type and timing of biliary drainage for acute cholangitis are determined by the severity of the clinical presentation as well as the availability and feasibility of drainage techniques^9^,^10^,^11^,^12^. Either an urgent endoscopic or percutaneous transhepatic biliary drainage should be performed as soon as possible to decompress the bile duct for the patients with severe acute cholangitis. Laparoscopic common bile duct exploration (LCBDE) which can drain bile duct and remove CBD stones is a well-established surgery procedure for the treatment of patients with CBD stones in the elective situations^13^. The safety and efficiency of LCBDE have been well-documented in the elective situations. However, it is yet to be elucidated the timing and approaches of LCBDE that should be employed in patients with CBD stone-related nonsevere (mild to moderate) acute cholangitis. Especially, the importance of emergent LCBDE in those patients has not been validated. Therefore, the purpose of this study is to compare the effects of early (≤72 h of admission) or delayed (>72 h of admission) LCBDE on the patients with CBD stone-related nonsevere acute cholangitis during emergent admissions.

## Materials and methods

This retrospective clinical study was conducted at the Laparoscopic Surgical Center of the Department of General Surgery and approved by institutional review board (IRB) committee at Beijing Shijitan Hospital of Capital Medical University. Informed consent was obtained from all subjects. This study was carried out in accordance with established national and institutional ethical guidelines regarding the involvement of human subjects and the use of human tissues for research. A total of 73 patients with the CBD stone-related nonsevere acute cholangitis and gallbladder stones were included from Jan 2007 to Dec 2014. All of them had received both LCBDE (choledochotomy) and laparoscopic cholecystectomy (LC) surgeries during emergent admission. The patients are divided into two groups according to the length from admission to surgery, i.e., early (≤72 h of admission, n = 32) and delayed (>72 h of admission, n = 41) LCBDE groups. All patients had a dilated CBD (>8 mm in diameter) with choledocholithiasis by magnetic resonance cholangiopancreatography (MRCP) and gallbladder stones as demonstrated by sonography before LCBDE. The diagnosis of nonsevere (mild to moderate severity) acute cholangitis was based on a combination of clinical features, as well as laboratory and imaging results. None of the patients had preoperative endoscopic retrograde cholangiopancreatography (ERCP). The medical records of 412 patients who had been diagnosed with acute cholangitis were reviewed during the period of time. Severe acute cholangitis with organ dysfunction was excluded^3^,^4^. The patients with previous upper abdominal surgery, obesity [body mass index (BMI) ≥35 kg/m^2^], acute pancreatitis, generalized peritonitis, untreated coagulopathy, severe cardiopulmonary diseases, advanced cirrhosis with hepatic dysfunction or other co-morbid conditions which preclude general anesthesia and operation were also excluded^13^. The patients with suspected malignant or other non-stone obstruction of bile duct were excluded in this study^14^. Among the 412 patients, 339 patients were excluded.

The demographic (age, sex) and clinical characteristics including the diameter of CBD, number of CBD stones, pathologic type of cholecystitis, duration of surgical procedure, conversion rate, complication rate, retained stone of bile duct, length of hospital stay (LOS), total charges, as well as the effect of LCBDE on the patients were compared between the two groups.

Once nonsevere acute cholangitis was diagnosed, broad spectrum intravenous antibiotics (Cephalosporin-based therapy + metronidazole) were administrated immediately and lasted for about 4 days after LCBDE.

LCBDE was performed randomly by two specialists with over 10 years of experience in hepatobiliary and laparoscopic surgery. The surgical duration were comparable between the 2 groups. Once the diagnosis of nonsevere acute cholangitis was made, if the patient's physical condition was allowed, the surgery was followed. The patients were assigned to the surgeons randomly. Normally, the standard four-trocar operative technique was used for LCBDE and LC. After the Calot’s triangle was clearly exposed, an incision was made on the CBD longitudinally. First, the CBD was irrigated using saline to remove the stones. Second, the choledochoscope with basket was used to remove the stones and to examine the remnant ones. If the stones cannot be removed by the ways mentioned above, the retained stones would be removed by choledochoscope postoperatively. Intraoperative choledochoscopic examination was not required in order to shorten the surgery time. If necessary, it had to be finished within 30 minutes. No patients underwent intraoperative cholangiography. The T-tube was used to all patients. A drain was left at foramen of Winslow for several days postoperatively. If there were no detectable stones by postoperative cholangiography, the patients would be discharged with T-tube clamped after several days’ recovery. However, if there were retained stones in the CBD, the patients would be discharged with the T-tube opened and clamped alternately. All of the patients were readmitted after 4–8 weeks. Cholangiography and/or choledochoscopy were required for all patients before T-tube was removed.

### Statistics

Statistic analysis was performed with SPSS 13.0 software (SPSS, Chicago, IL). Continuous data were compared using the Student t-test. Categorical data were compared with the Pearson χ^2^ test. A *p* value < 0.05 was considered statistically significant.

## Results

The characteristics of patients in early and delayed LCBDE groups were listed in Table 1. The patients in the two groups were comparable in age and sex. 32 patients (43.8%) received early LCBDE and 41 patients (56.2%) received delayed LCBDE. There is no significant difference in either the diameter of CBD between groups by comparing the imaging data and the findings during surgery. There was no significant difference with regard to the number of CBD stones, acute / chronic cholecystitis and duration of the surgery. There was no conversion to open surgery, no major bile duct injuries and no deaths in either group.

One case was readmitted because of attack of coronary artery disease 1 week later. Overall, 3 and 5 patients in early and delayed LCBDE group, respectively, had retained stones, which were discovered by postoperative cholangiography and/or choledochoscopy. Three patients in each group developed bile leak, which was conservatively cured by drainage. There was no significant difference in the postoperative hospital stay between the two groups (11.91 ± 5.78 vs. 10.33 ± 8.09, *P* > 0.05). However, significant difference was observed in the LOS (13.34 ± 4.48 vs.18.32 ± 9.13, *P* < 0.05) and total costs (17712 ± 5446.63 vs. 21776 ± 7230.41 ¥RMB, *P* < 0.05) between the early and delayed LCBDE groups. The retained stones of those 8 patients were removed by choledochoscopy before T-tube was removed. No patients developed stricture of the CBD after LCBDE during the follow-up period. Septic symptoms were eliminated after LCBDE in all patients. Abdominal pain, jaundice, and laboratory data were improved as well (Table 2,3).

## Discussion

Acute cholangitis is one of the most serious complications of CBD stones. Therefore, safe and effective therapeutic approaches are urgently needed. Approximately 80% of patients with acute cholangitis respond to broad spectrum antibiotics, whereas the rest requires early biliary drainage, including the patients with nonsevere acute cholangitis^15^. Enodoscopic drainage followed by LC is a well-established treatment for severe acute cholangitis. We also offered ERCP to patients with high-risk of surgery and severe acute cholangitis. The alternative approaches for patients with nonsevere acute cholangitis are either ERCP/ endoscopic biliary sphincterotomy (EST) followed by LC or LCBDE and LC after control of acute cholangitis with antibiotics. Unfortunately, some patients being managed conservatively progress to sepsis or shock, succumb to a high morbidity and mortality, and thus require emergent drainage of biliary ductal system because of treatment failure. Tokyo Guidelines recommends that endoscopic drainage should be first-choice treatment for acute cholangitis^16^. Recently, Jang *et al.* demonstrated that urgent ERCP is the proper approach for the management of patients with CBD stone-related mild to moderate acute cholangitis due to the safety and short hospital stay^15^. Our results of emergent LCBDE for nonsevere acute cholangitis were similar to theirs of urgent ERCP.

ERCP/ EST for stone extraction is an optional approach which may be performed before, during or after LC with similar morbidity and mortality as well as clearance rates as that with LCBDE in the elective situations^17^. However, unless performed intraoperatively during cholecystectomy, ERCP/EST requires at least one additional procedure for cholecystectomy. Moreover, it damages Oddi sphincter and may cause complications, such as pancreatitis, bleeding, and duodenal perforation. The combined LC with intraoperative endoscopic approaches with CBD stone extraction could be performed at one-stage procedure^18^. However, it may prolong surgery time and requires additional equipment and skills. Moreover, it has limited eligibility under the situations of stone impaction, gastrectomy or Roux-en-y anatomy, recurrent bile duct stones after prior open exploration of the CBD as well as biliodigestive anastomosis, periampullary diverticula, and Mirizzi syndrome^13^,^19^,^20^. Although LCBDE has been used for the treatment of patients with CBD stones, it has not been included in the clinical guidelines for the treatment of patients with acute cholangitis. Gholipour *et al.* showed that LCBDE is a more effective procedure as the initial modality of management for acute cholangitis with gallstone compared with open surgery^21^. However, it remains to be elucidated about the benefit of urgent LCBDE in the management of CBD stone-related nonsevere acute cholangitis.

In the current study, we found that early LCBDE is as safe and effective as delayed LCBDE for the treatment of CBD stone-related nonsevere acute cholangitis. Control of septic symptoms was achieved in all patients by LCBDE during emergent admissions. LCBDE with T-tube drainage is preferable and safer if the CBD is inflamed^13^. We followed this principle with T-tube drainage. In our study, 8.2% (6/73) of patients developed bile leak by LCBDE, which was higher than elective situations^22^. It might be associated with inflammation of CBD, while it was easily cured by drainage and did not require further procedures. The zero mortality in our study may be attributed to the strict criteria for patient enrollment and exclusion, with no severe cases being included and no conversion to open CBD exploration. The limitation of the current study is a relatively small sample size and lack of patients with very severe complications. Therefore, a large cohort study and randomized controlled trials (RCT) are needed to validate our findings.

According to the “Tokyo guidelines”, the biliary drainage should be performed as soon as possible for patients with moderate and severe cholangitis. It could be performed within 24 to 48 hours for patients with mild cholangitis^23^. However, most of the patients in the current study did not receive LCBDE within 24 to 48 hours after emergent admission due to the preoperative preparation and/or the co-morbidities which required consultation with other specialists for the safety of the operation. In fact, the septic symptoms of some patients were improved with conservative treatment before surgery. In our study, only 15.1% (11/73) and 26.0% (19/73) of patients received LCBDE within 24 and 48 hours of emergent admissions, respectively; and thus, 43.8% (32/73) patients received LCBDE within 72 hours of emergent admissions. In order to minimize the bias caused by different time-points of sampling, we artificially divided the patients into two groups according to the length from admission to surgery, i.e., early (≤72 h of admission, n = 32) and delayed (>72 h of admission, n = 41) LCBDE groups. Early LCBDE could shorten LOS and save expense compared to the delayed LCBDE, due to the shorter hospital stay and conservative treatment before surgery. Collectively, the LCBDE should be performed as soon as possible if the patients are subjective to surgery criteria and are able to tolerate general anesthesia and LCBDE.

ERCP/EST and LCBDE with or without stone extraction are probably compensatory therapeutic procedures for the treatment of acute cholangitis with CBD stones during emergent admissions. ERCP/EST is probably more appropriate for severe acute cholangitis and for patients with high-risk of surgery^16^. The patients with nonsevere acute cholangitis may benefit from LCBDE. The gallbladder and CBD stones may be taken care of simultaneously in a minimally invasive manner in order to keep the function of the sphincter and to avoid unnecessary second admission for delayed LC. LCBDE for emergent patients is relatively difficult, which requires skilled and experienced surgeons. The use of intraoperative choledochoscopy and cholangiography has reduced the retained stone rate to less than 5% for CBD stones in elective situations^24^,^25^. In our study, 11.0% (8/73) of retained CBD stones after LCBDE of nonsevere acute cholangitis is higher than elective situations, but LCBDE with T-tube drainage is safe and effective for the treatment of nonsevere acute cholangitis. The combination of cholangiography and choledochoscopy ensures clearance of the CBD postoperatively. Strictly speaking, removing retained stones should count as one operation in the postoperative period, but cholangiography and/or choledochoscopy were usually required for all patients before T-tube was removed in order to reduce the incidence of retained stones. If there were retained stones in the CBD, they were simultaneously removed by choledochoscopy before T-tube was removed.

In conclusion, both early and delayed LCBDE are safe and effective for the treatment of CBD stone-related nonsevere acute cholangitis during emergent admissions. Early LCBDE is recommended as it tends to shorten the LOS and costs less due to the shorter hospital stay before surgery. A large cohort study and RCT are needed in order to further validate our results.

## Additional Information

**How to cite this article**: Zhu, B. *et al.* Early versus delayed laparoscopic common bile duct exploration for common bile duct stone-related nonsevere acute cholangitis. *Sci. Rep.* **5**, 11748; doi: 10.1038/srep11748 (2015).

## Figures and Tables

**Table 1:** Comparison of characteristics between early and delayed LCBDE group.

**Demographic data/Clinical Parameters**	**Early LCBDE (n = 32)**	**Delayed LCBDE (n = 41)**	P*
Age (years), median ± SD	59.06 ± 15.72	59.85 ± 14.6	0.841
Sex (Male / Female )	15/17	20/21	0.872
Clinical context and manifestations, n (%)			
History of biliary disease	19 (59.4)	22 (53.7)	0.625
Fever and/or chills	19 (59.4)	24 (58.5)	0.942
Jaundice	18 (56.3)	24 (58.5)	0.845
Abdominal pain (RUQ or upper abdominal)	31 (96.9)	39 (95.1)	0.708
Imaging and/or intra-operative findings			
Diameter of common bile duct (mm)	11.34 ± 3.19	12.93 ± 4.63	0.096
No. of solitary CBDS, n (%)	10 (31.3)	14 (34.1)	0.794
No. of multiple CBDS, n (%)	22 (68.8)	27 (65.9)	
Pathologic type of gallbladder			
Acute cholecystitis, n (%)	6 (18.8)	11(26.8)	0.418
Chronic cholecystitis, n (%)	26 (81.3)	30 (73.2)	
Duration of surgery (min), median ± SD	110.78 ± 48.63	118.90 ± 59.97	0.525
Complications, n (%)			
Pulmonary infection	1 (3.1)	0 (0)	0.438
Retained stone	3 (9.4)	5 (12.2)	0.702
Bile leak	3 (9.4)	3 (7.3)	0.751
30-day admission, n (%)	1 (3.1)	0 (0)	0.438
LOS (days), median ± SD	13.34 ± 4.48	18.32 ± 9.13	0.006
Postoperative hospital stay (days), median ± SD	11.91 ± 5.78	10.33 ± 8.09	0.355
Total cost (Yuan in RMB), median ± SD	17712 ± 5446.63	21776 ± 7230.41	0.031

RUQ, right upper quadrant; CBDS, common bile duct stone; LOS, length of hospital stay.

^*^P value was obtained from Pearson χ^2^ test and independent-samples t-test.

**Table 2:** **Comparison of outcomes in early LCBDE patients before and after surgery.**

**Variable**	**Before early LCBDE (n = 32)**	**After early LCBDE (n = 32)**	P*
Laboratory data (median ± SD)			
WBC count (×10^9^/L)	11.46 ± 2.93	8.84 ± 4.14	0.005
ALT (U/L)	173.84 ± 97.85	56.18 ± 39.67	0.000
AST (U/L)	144.96 ± 87.94	33.87 ± 20.03	0.000
Total bilirubin (μmol/L)	66.45 ± 40.37	27.50 ± 12.55	0.000
ALP (U/L)	178.28 ± 120.23	121.75 ± 84.64	0.000
γ-GTP (GGT) (U/L)	366.66 ± 211.26	195.72 ± 116.21	0.000

WBC, white blood cell; ALT, alanine aminotransferase; AST, aspartate aminotransferase; ALP, alkaline phosphatase; γ-GTP (GGT), γ-glutamyl transferase (gamma-glutamyl transpeptidase).

^*^P value was obtained from Paired-samples t-test.

**Table 3:** **Comparison of outcomes in delayed LCBDE patients before and after surgery.**

**Variable**	**Before delayed LCBDE (n = 41)**	**After delayed LCBDE (n = 41)**	P*
Laboratory data (median ± SD)			
WBC count (×10^9^/L)	10.91 ± 2.63	8.54 ± 3.87	0.001
ALT (U/L)	155.17 ± 91.87	45.49 ± 29.38	0.000
AST (U/L)	130.68 ± 91.33	31.02 ± 15.35	0.000
Total bilirubin (μmol/L)	59.99 ± 41.91	31.50 ± 16.61	0.000
ALP (U/L)	206.66 ± 96.13	127.15 ± 61.61	0.000
γ-GTP (GGT) (U/L)	435.10 ± 246.89	203.90 ± 157.08	0.000

^*^P value was obtained from Paired-samples t-test.
